# First Strike: Description of the Events at the First Salmon Farm Affected by the 2025 Algal Bloom in Northern Norway

**DOI:** 10.1111/jfd.70162

**Published:** 2026-03-15

**Authors:** Julie Seem, Mirjam Nikoline Petterson, Tore Seternes, Pål Furset Lader, Julie Christine Svendsen, Lars‐Johan Naustvoll, Lars Helge Stien

**Affiliations:** ^1^ Nordlaks Havbruk AS Stokmarknes Norway; ^2^ Norwegian College of Fishery Science, Faculty of Biosciences, Fisheries and Economics UiT The Arctic University of Norway Tromsø Norway; ^3^ Department of Marine Technology Norwegian University of Science and Technology (NTNU) Trondheim Norway; ^4^ Department of Aquatic Animal Health and Welfare The Norwegian Veterinary Institute Ås Norway; ^5^ Plankton Research Group Institute of Marine Research Bergen Norway; ^6^ Animal Welfare Research Group Institute of Marine Research Bergen Norway

**Keywords:** acute mortality, Atlantic salmon, *Chrysochromulina*, contingency plan, harmful algal bloom (HAB), *Phaeocystis*

## Abstract

Harmful algal blooms (HABs) are a threat to fish welfare, occurring suddenly and unexpectedly causing significant consequences for fish and salmon farmers worldwide. Norwegian farmers have been facing this challenge at irregular intervals since the very beginning of the industry. This report describes the events on the first fish farm affected by the spring HAB of 2025 in northern Norway. Specifically at the production site Fornes, which at the time of the strike had more than 1 million Atlantic salmon (*Salmo salar*) with an average weight above 3 kg in its sea cages. First signs of the event were reduced fish appetite and cloudy water appearance, followed by changes in fish behaviour and acute mortality of 39.5%. Water samples showed dominance of the phytoplankton species *Phaeocystis pouchetii*, followed by *Chrysochromulina leadbeateri*. Necropsy of the fish and histopathological lesions on the gills and liver supported that the mortality was caused by the algae. The farm's contingency plan was enforced immediately when the acute high mortality was observed. Including, but not limited to, feed withdrawal followed by emergency harvest of the whole production site. The dramatic incidence at Fornes highlights the urgent need for monitoring and early‐warning systems for HABs, as well as further development of suitable mitigation strategies and contingency plans to minimise the effect of HABs once they have been forecasted or detected.

## 1 Introduction

Harmful algal blooms (HABs) are a threat to farmed Atlantic salmon ( *Salmo salar* ) (hereafter named salmon) worldwide. Incidences where blooms have resulted in significant amounts of mortalities, in some cases thousands of tonnes, of farmed salmon are reported from countries such as Chile (Apablaza et al. 2017), Canada (Martin et al. 2006), New Zealand (Chang et al. 1990; MacKenzie et al. 2011), Scotland (Treasurer et al. 2003), Sweden (Berge et al. 1988), Denmark (Moestrup 1994) and Norway (Rey and Aure 1991; Karlson et al. 2021; Fon 2025). The reasons why HABs cause mortality depends on the species involved and the characteristics of the bloom. Some algae can cause pathological lesions on the gills due to toxicity or from direct physical damage (Rodger et al. 2011). Other reasons for mortality include oxygen supersaturation, oxygen depletion and physical clogging of the gills (Burkholder 1998; Rodger et al. 2011).

HABs have been a risk factor in Norwegian salmon farming from the very beginning. Already in its infancy in 1981, a bloom of *Gyrodinium aureolum* caused mortality in farmed salmon along the south coast of Norway (Dahl and Tangen 1999). Since then, there have been several HABs with a variety of different algae. *Prymnesium polylepis* caused mortality of approximately 800 tonnes (t) of farmed salmon along the south‐ and western coasts of Norway in 1988 (Berge et al. 1988; Karlson et al. 2021). *Prymnesium parvum* caused mortalities of around 750 t of salmon on the southwestern coast of Norway in 1989, as well as 135 t in 2007 (Johnsen and Lein 1989; Johnsen et al. 2010; Karlson et al. 2021). *Chrysochromulina leadbeateri* caused mortalities of around 742 t of farmed salmon in Northern Norway in 1991 (Rey and Aure 1991; Karlson et al. 2021), and *Pseudochatonella* sp. caused mortality in 2024 (Naustvoll 2025b). The latter algal species also had significant blooms along the south coast in 1998 and in 2001 with 350 t and 1100 t of salmon lost and some lesser blooms in 2006 and 2007 (Karlson et al. 2021), *Alexandrium tamarense* caused mortality of around 800 t at a farm in Northern Norway in 2014 (Gardar 2014). Although this is probably not a complete list, the sporadic nature of the blooms and the long time since the last really large bloom that affected more than isolated farms led to a sense of security and that the big threats from algal blooms were a thing of the past in the late 2010s (Winther 2018). The spring of 2019 did, however, see the worst algal bloom so far, with *C. leadbeateri* killing 13,400 t (7.5 million fish) of farmed salmon in Northern Norway (Karlsen et al. 2019; Fon 2025).

Both the 1991 and the 2019 bloom of *C. leadbeateri* happened in Northern Norway, more precisely in the fjords surrounding the Lofoten and Vesterålen islands. The HAB in 1991 took place between the 16th of May and 20th of June, whereas the bloom in 2019 happened between the 5th of May and 6th of June. Environmental conditions preceding the HAB events were characterised by mild winters and high freshwater runoff, the latter resulting in lower salinity and stratification of the water column. The days leading up to the blooms were characterised by sunny weather and little wind. Additionally, high amounts of available nutrients in the fjords might have favoured the growth of *C. leadbeateri* (Karlsen et al. 2019; Fon 2025). During the winters prior to the bloom in 1991, approximately 1 million tonnes of herring were overwintering in the fjord, leading to depletion of oxygen and excessive amounts of biological waste, which might have contributed to the increased phytoplankton production. Other hypotheses of the source of nutrients were nitrogenous waste from the sediments underneath the cages and nutrients arriving with freshwater runoff (Rey and Aure 1991). The latter was also mentioned as a contributing factor for the bloom in 2019 (John et al. 2022). It was typically farms with large salmon or newly transferred smolt that were most affected, and there was also a trend towards higher mortality in sea cages with high fish densities (Rey and Aure 1991). Newly transferred smolt are at a critical life stage where they are vulnerable to additional stressors (Iversen et al. 2005; Tvete et al. 2023). Similarly, large salmon are known to be more vulnerable to hypoxia (Hvas et al. 2025), and it is also well known that high fish density increases the risk of hypoxic conditions inside a sea cage (Johansson et al. 2006). Investigations in the aftermath of the 2019 HAB found that the toxins from *C. leadbeateri* have the potential to harm the gills of farmed salmon (Wang et al. 2024; Fon 2025).

During the spring of 2025, a new HAB happened in the fjords around Lofoten and Vesterålen in Northern Norway. This time with two dominating algal species, *Phaeocystis pouchetii*, followed by *C. leadbeateri* . This article describes the sequence of events at the first salmon farm affected by this bloom.

## 2 The Algal Bloom at the Production Site Fornes

### 2.1 The Farm Site

**FIGURE 1:**
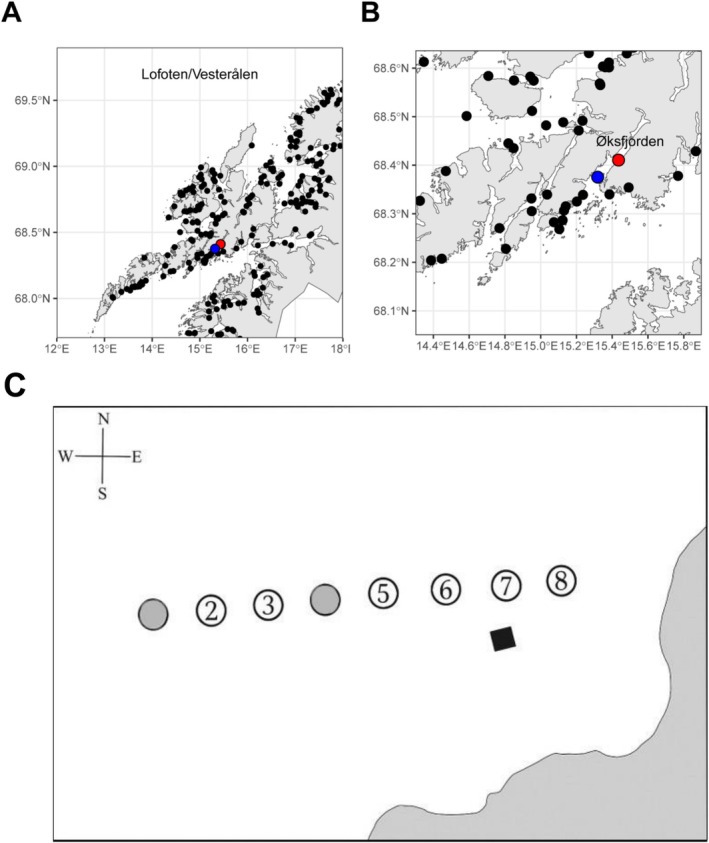
Geographical area of the salmon farm Fornes. The red dot in A and B is the location of Fornes, the blue dots shows the location of a nearby farm, Hallvardøy and the black dots illustrate other salmon farms in the area (Norwegian Directorate of Fisheries, n.d.‐b). (A) A map of Vesterålen and Lofoten. (B) A map zoomed in on Øksfjord. (C) The layout of the farm. There were fish in cage 2, 3, 5, 6, 7 and 8 while cage 1 and 4 were not in use.

The production site Fornes is located at 68.410° N, 15.435° E in Øksfjorden (Figure 1), a branch of Vestfjord located in Lødingen municipality, Nordland County, Norway (Thorsnæs 2024; Synvis 2025; Norwegian Directorate of Fisheries n.d.‐a).

As part of the farm company's mapping of the production site's characteristics, the water current was measured between the 19th of July and 5th of November 2024 (Synvis 2025). The measurements were performed with an acoustic doppler current profiler (Aquadopp Profiler, Nortek) by Sea Eco AS and showed the main current direction of particle transport was towards southeast (230 degrees) with a returning current towards northwest (35°), measured at 86 m depth. The average current speed was 9 cm s^−1^ and the maximum current speed was 28.9 cm s^−1^.

The sea floor is gradually getting deeper away from land and reaches 58–126 m underneath the production site and over 200 m centrally in the fjord (Lippestad 2024).

There were in total six cages with salmon at Fornes at the time of the algal bloom, placed in cage 2, 3, 5, 6, 7, and 8, while cage 1 and 4 were not in use (Figure 1C). The size of the cages was 160 m in circumference and had cone‐shaped nets extending 55 m deep. Prior to the incident, all cages were equipped with semi‐permeable skirts down to 10 m. These were removed on the 26th of April.

### 2.2 The Fish

The salmon at Fornes all originated from the same egg batch and were reared in the same hatchery during the same time period. Since the algae bloom affected all cages equally, the following sections will include data from the production site in total and not separate cages. On the 19th of April there were in total 1,018,192 fish with an average weight of 3379 g at the production site, which equals to 3,441 t.

Earlier diagnoses of the fish were the viral diseases Infectious Pancreatic Necrosis Virus (IPNV) and Heart and skeletal muscle inflammation (HSMI), which were diagnosed mainly during the months after sea transfer in April–May 2024. Complex gill disease (CGD) was diagnosed during the following autumn, whereas winter ulcers caused by the bacterium *Moritella viscosa* were the dominating cause for mortality during the winter months. However, the daily mortality had been below 0.009% for the 4 months preceding the HAB, a level classified as Level 0 according to the Laksvel operational welfare monitoring protocol (Level 0 = daily mortality below 0.01%) (Nilsson et al. 2025).

On farms with over one million fish, veterinarians or fish health biologists are required to visit at least 12 times per year (Akvakulturdriftforskriften 2008), with each visit producing a detailed health report documenting the fish's condition and welfare. According to these monthly assessments, the salmon at Fornes were reported to be in good health and welfare in the weeks leading up to the HAB.

### 2.3 The Environment

The winter of 2024–2025 was generally warmer than the previous winter in the region (Lusedata 2026). These months were also characterised by several windy and stormy days; however, there was little wind (Meteorologisk institutt (MET) 2026) and sunny weather (personal comment, 2025) the week prior to the bloom.

**FIGURE 2:**
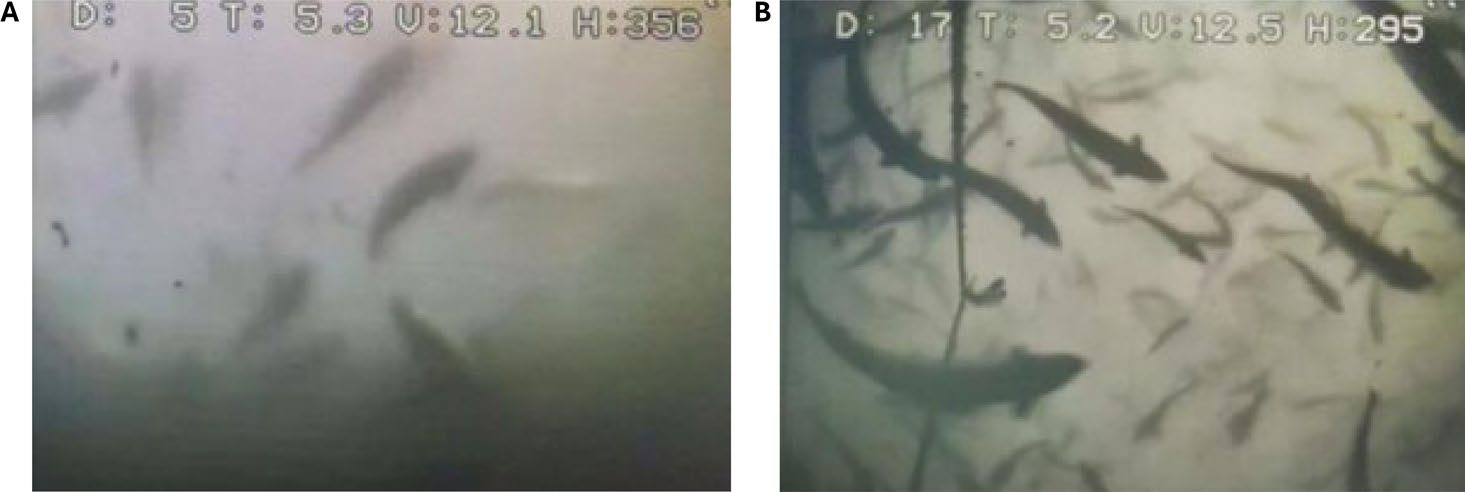
Image of the milky‐ and turbid water at Fornes, captured on the 26th of April. (A) At 5 m depth. (B) At 17 m depth. Photo: Stian Hagan, Nordlaks.

In the light of the event, observations of water appearance were made by fish health and farm personnel at the production site, as well as by operators at the feeding centre monitoring the salmon's appetite via remote‐controlled underwater cameras (Figure 2). From these observations, a milky colour and consistency of the water was first reported on the 24th of April and persisted in the following days. These observations were sporadic and descriptive, rather than part of a systematic measurement, but they help illustrate the environmental conditions during the event.

#### 2.3.1 Oxygen and Temperature

Oxygen and temperature were recorded with an Orbit‐3650 camera (ScaleAQ) at 5 m depth at the production site Fornes approximately at 8 am every day. Due to practical considerations around preparing the cages for emergency harvest of the fish, the camera was removed on the 27th of April. A nearby salmon farm in the same fjord (Figure 1A,B) measured the temperature and oxygen saturation at 5 m depth in the same time period with shorter time intervals (every hour) using an Aqua Optode 4‐20 mA (Aanderaa, Xylem). This production site, Hallvardøy, was also affected by the HAB a few days after Fornes.

**FIGURE 3:**
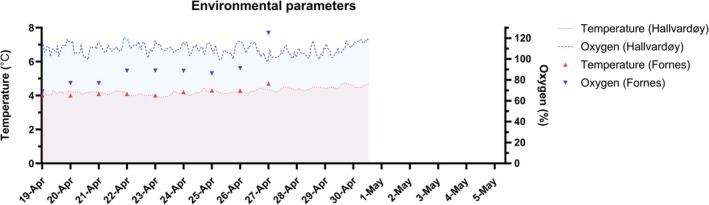
The temperature(°C) and oxygen saturation (%) at Fornes and Hallvardøy at 5 m depth between 19th of April and 5th of May. The temperature (red) and oxygen (blue) were measured once a day at Fornes, marked with a triangle. Measurements of temperature (red) and oxygen (blue) was measured every hour at Hallvardøy, illustrated with a dotted line and a shaded area below the curve.

The temperature recorded at the two production sites closely followed each other (Figure 3). The graph shows a different trend with the oxygen saturation, which is higher throughout most of the period at Hallvardøy compared to Fornes. This can be explained by the placement of the sensors. At Fornes, a camera was used which was placed closer to the school of fish while the environmental sensor at Hallvardøy was placed further away from the fish. The temperature and oxygen saturation at Fornes gradually increased day by day from 4°C to 4.7°C and 69% to 125%, respectively (Figure 3). On the 30th of April, preparations for harvesting started also at Hallvardøy and we therefore do not have temperature and oxygen data beyond this time point.

### 2.4 Water Samples of *Phaeocystis* and *Chrysochromulina*

Water samples were collected with a water collector (Ruttner, standard water sampler) at 2‐, 4‐, and 8‐m depth on the 26th and 29th of April and sent to SeaEco AS in Harstad, Norway, for analysis. The sampling point was on the north side of sea cage 8, sampling the in‐flow water to the production site.

The samples collected on the 26th of April showed high densities of *P. pouchetii* at all three depths and the highest concentrations were observed at 2‐ and 4‐m depth (Table 1). The algae were present mainly as large colony forms; however, conservations of the samples resulted in the majority of the colonies disintegrating, and the counts were mostly sickle cells of *P. pouchetii* . *Heterosigma akashiwo* was detected in low densities, similar in numbers to *C. leadbeateri* , *Chaetoceros* spp., and other Prymnesiales (Table 1).

**TABLE 1:** Results from water samples collected at three depts (2 m, 4 m and 8 m) on the 26th and 29th of April at the affected production site.

Species	25th of April			29th of April		
	2 m	4 m	8 m	2 m	4 m	8 m
Prymnesiales	—	8289	—	—	—	—
*Chrysochromulina* spp.	16,578	33,156	16,578	1,012,605	953,040	—
*Phaeocystis* spp, cells	4,592,106	4,409,748	1,798,713	1,250,865	2,334,948	—
*Heterosigma akashiwo*	16,578	8289	—	—	—	—
*Chaetoceros* spp. > 10 my	47,013	8289	—	51,161	—	—
*Chaetoceros* spp. < 10 my	82,890	33,156	—	27,655	—	—
Total (cells/L)	4,755,165	4,500,927	1,815,291	2,349,200	3,287,988	0

*Note:* The concentration of algae is given as cells pr litre (cells/L). “‐” indicates the species was not detected in the sample.

**FIGURE 4:**
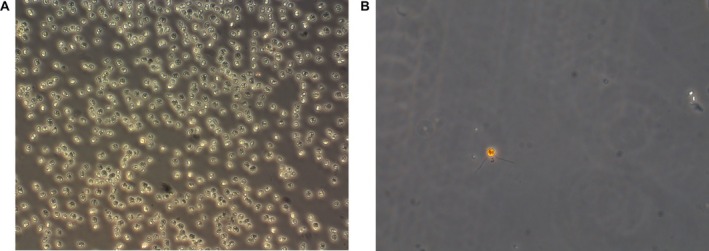
(A) Single cells of a *Phaeocystis pouchetii* colony (B) *C. leadbeateri* . Water samples were fixed with Lugol's solution and the image was captured using a light microscope at 400× magnification. *Photo*: Institute of Marine Research, algae lab.

In the water samples collected on the 29th of April, high densities of *P. pouchetii* and *C. leadbeateri* were detected both at 2 and 4 m (Table 1). In addition to lower concentrations of *Chaetoceros* spp. (< 10 my) at 2 m depth compared to the samples collected on the 26th (Table 1). Figure 4 presents the algae *P. pouchetii* and *C. leadbeateri* in a water sample.

### 2.5 Fish Appetite and Daily Feed Output

The feeding regime at the farm was appetite‐feeding once a day. Meaning that dedicated personnel at a central feeding office monitored the underwater cameras in each cage observing the behavioural response of the fish to the feed. When the fish exhibited low response to the feed, the meal was complete. Daily feed output thus becomes an indirect measurement of fish appetite.

**FIGURE 5:**
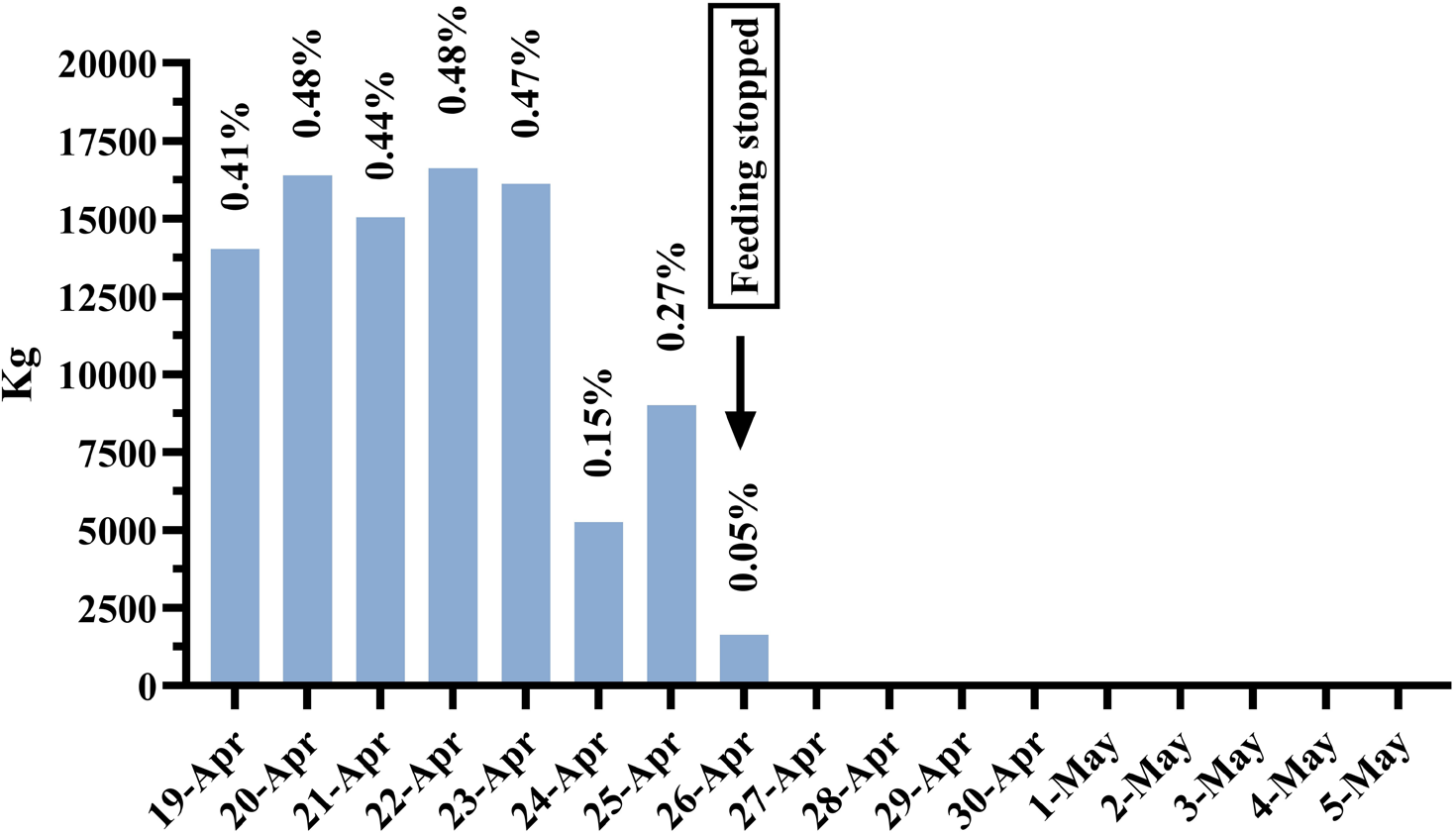
Daily feed output (kg) and the specific feeding rate per day (% day^−1^) calculated as (kg feed per day/kg fish) × 100, at the production site between 19th of April and 5th of May. Feeding was stopped on the 26th of April.

Prior to the algal bloom, the daily specific feeding rate ((kg feed per day/kg fish) × 100) was ranging between 0.41% and 0.48% day^−1^ (Figure 5). Contrary, on the 24th of April, the appetite and feeding rate was dramatically decreased to 0.15% day^−1^, which was only about one third compared to the day before. The fish also exhibited a low response to the feed and swam deeper than usual (observations by the feed operators). These observations persisted in the following days. On the 25th of April, there was a slight increase in the daily feed output before feed was fully withdrawn during the morning of the 26th of April, when the operators observed increased mortality in all cages at the production site.

### 2.6 Fish Behaviour

In the fish health reports from the past 4 months prior to the HAB, the fish at Fornes was described as having normal behaviour in all cages, including a stable and well‐coordinated schooling behaviour and exhibited a good flight response to visual provoking stimuli.

The responsible feeding operators, stationed at the feeding center, reported no deviations in behavior seen on the underwater cameras prior to the incident. Notably, on the 24th of April, the fish were swimming deeper than usual and exhibited decreased appetite in all cages. In addition, the number of fish passing the cameras was lower than usual, indicating reduced swimming activity.

The farm personnel working alongside the fish did not see any signs of unusual or deviant behavior on the 24th of April. Interestingly, on the 25th of April, there was a slight increase in individuals swimming along the net wall, some with decreased response to visual stimuli. Waving with hands over the water surface and shaking the net pen wall did not cause the fish to swim away as fast as expected.

Fish health personnel visited Fornes for an acute health visit on the 26th of April, in response to the onset of acute mortality. When observing the fish around the cage, there was an absence of flight response and reactions to visual stimuli. In addition, the fish exhibited a slow swimming pattern with apathic behavior and gasping at the surface.

**FIGURE 6:**
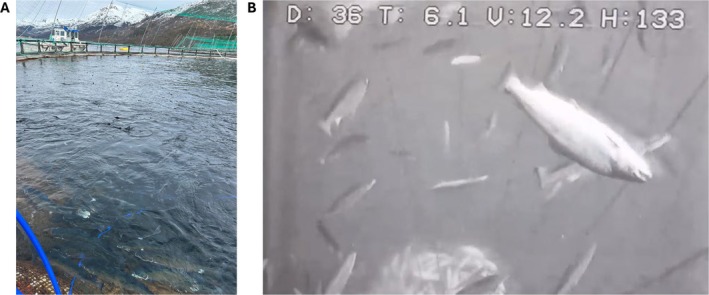
Pictures taken on the 26th of April. (A) Image of fish in one of the affected cages. High number of fins visible on the surface of the water in addition to some apathic individuals in the lower part of the picture. *Photo*: Stian Sjaastad, Nordlaks. (B) Several fish losing equilibrium before gradually sinking to the bottom of the cage, captured at 36 m deep with an underwater camera. *Photo*: Stian Hagan, Nordlaks.

On the same date, operators working at the production site reported a high number of moribund fish in the upper layers of the water column (Figure 6A). With the help of an ROV‐camera, they observed that the fish which were heavily influenced by the algal bloom were swimming in the upper water levels, tightly to each other and with apathetic behaviour. However, below 20–25 m, the fish looked healthier, although they did not swim in a circular pattern, but rather back and forth and seemed stressed. When pending the camera up and down in the sea cages, individual fish along the water column and the net wall were observed losing equilibrium and sinking towards the bottom of the cage (Figure 6B).

Farm personnel also commented on observations of wild fish living outside the cage with abnormal behavior, including repeated collisions toward the net of the cages.

### 2.7 Fish Mortality and Implementation of Emergency Plan

The daily mortality percentage at the production site during the last 4 months prior to the algal bloom ranged between 0.003–0.009%. On the 26th of April, a dramatic increase in mortality was observed in all cages. The acute high mortality persisted in the following days and between the 25th of April and 5th of May the total accumulated mortality at the production site was 39.47% out of 1,017,981 fish, which equals 401,794 individual fish and 1360 t.

The farm's emergency plan was put into force when acute high mortality was first revealed and all possible service‐ and working boats, ensilage boats and well boats in the area were gathered to manage the situation. See Figure A1 to get an impression of the scale of the operation. Feed withdrawal was the first measure implemented on the 26th of April in order to lower the metabolic rate, reduce the oxygen demand of the fish, and lower the surface activity of the population (Dale et al. 2022; Hvas et al. 2024). In this particular case, where the highest densities of the algae were detected in the upper layers of the water column, it most likely had a positive effect by avoiding the bloom and redirecting the fish deeper in the water column. Thereafter, the lice skirts were removed. Several studies have documented that lice skirts can have a lice‐reducing effect (Lind et al. 2015; Volent et al. 2020). On the other hand, studies have also shown that the oxygen saturation often is lower inside cages with skirts compared to cages without skirts (Lind et al. 2015; Jónsdóttir et al. 2023). Regarding protection against algae, results vary from high protection to no protection at all, depending on several factors such as characteristics of the algal species and the environmental condition at the farm (Volent et al. 2020; Dale et al. 2022). In this particular case, the lice skirts were removed with the intention of increasing the flow of water through the cages, improving the environmental conditions for the suffering fish.

Several options were discussed for the fish at Fornes, based on available measures. One possibility was to continue rearing the fish at the production site, hoping for the HAB to pass. The second option was to move the fish to another location, unaffected by the HAB. The third possibility was to harvest the fish right away. Based on the prognosis of the fish at Fornes evaluated by fish health personnel, the latter option was put into force. On the 29th of April, the first shipment of fish was transported to the harvest plant. The other cages were harvested as rapidly as possible until the production site was empty of fish on the 5th of May. In total, 656,934 fish with an average weight of 3436 g were emergency harvested, which equals 2257 t.

**FIGURE 7:**
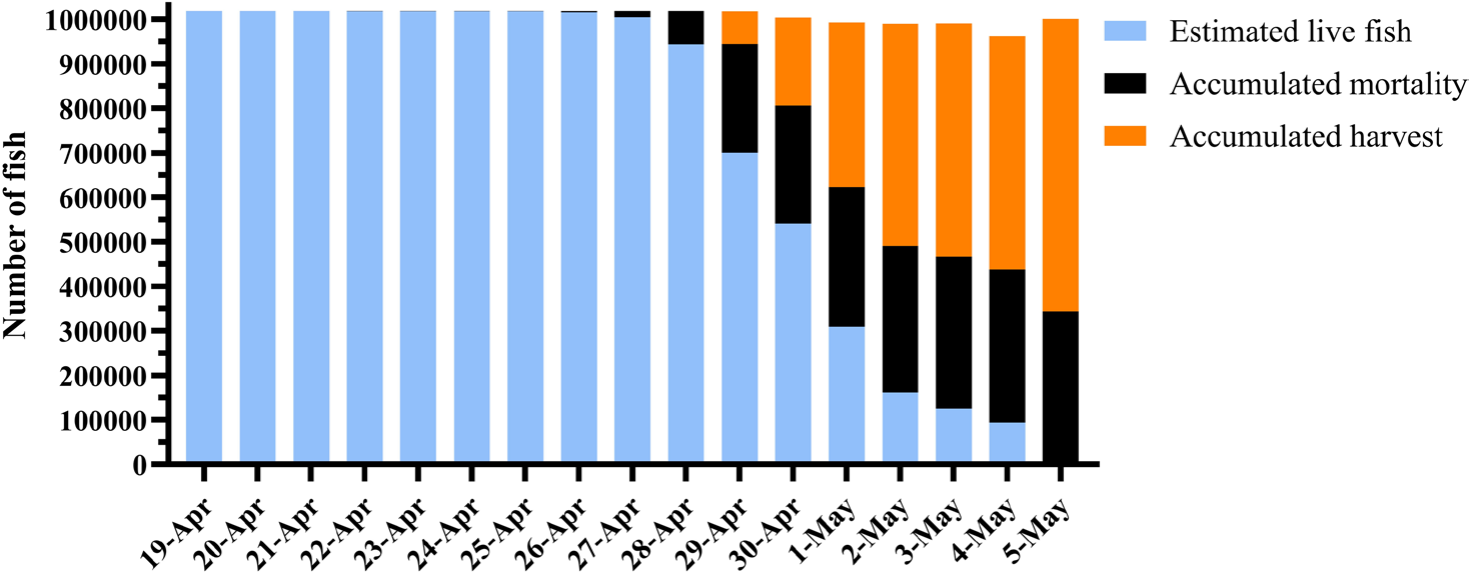
Registered daily progression at Fornes, including the number of estimated live fish at the farm (blue), accumulated registered mortality (black) and accumulated registered harvested fish (orange) between 19th of April and 5th of May. The number of fish categorised under accumulated mortality and accumulated harvest is expressed as accumulated over time whereas estimated live fish is expressed as the actual number on the respective day.

Due to acute high mortality in all cages at the same time and capacity limitations on ensilage‐ and working boats, the number of fish collected and registered varied between the dates and the number of estimated live fish displayed in the graph (Figure 7) does not precisely describe the situation with regards to the actual daily mortality. For example, on the 26th of April, a high number of dead fish was observed with the help of a feeding camera, but due to technical problems and limitations on ensilage boats, only a small fraction of the dead fish was collected on that date; the remaining were instead collected on the 27th of April, or even on the 28th. However, the last pole in the graph, on the 5th of May, shows the total extent of the HAB, displaying the accumulated mortality and accumulated harvested fish from start till end of the production. In addition to this, the total number of fish in each pole in the graph has some daily variations between the 30th of April and 5th of May; this is due to deviations of quantifying the fish in this acute situation. The ensilage boats calculate the number of tonnes received in the boat and not the exact number of fish. And there were some variations between the size of the fish within the cage and between cages.

### 2.8 Necropsy of the Fish

**FIGURE 8:**
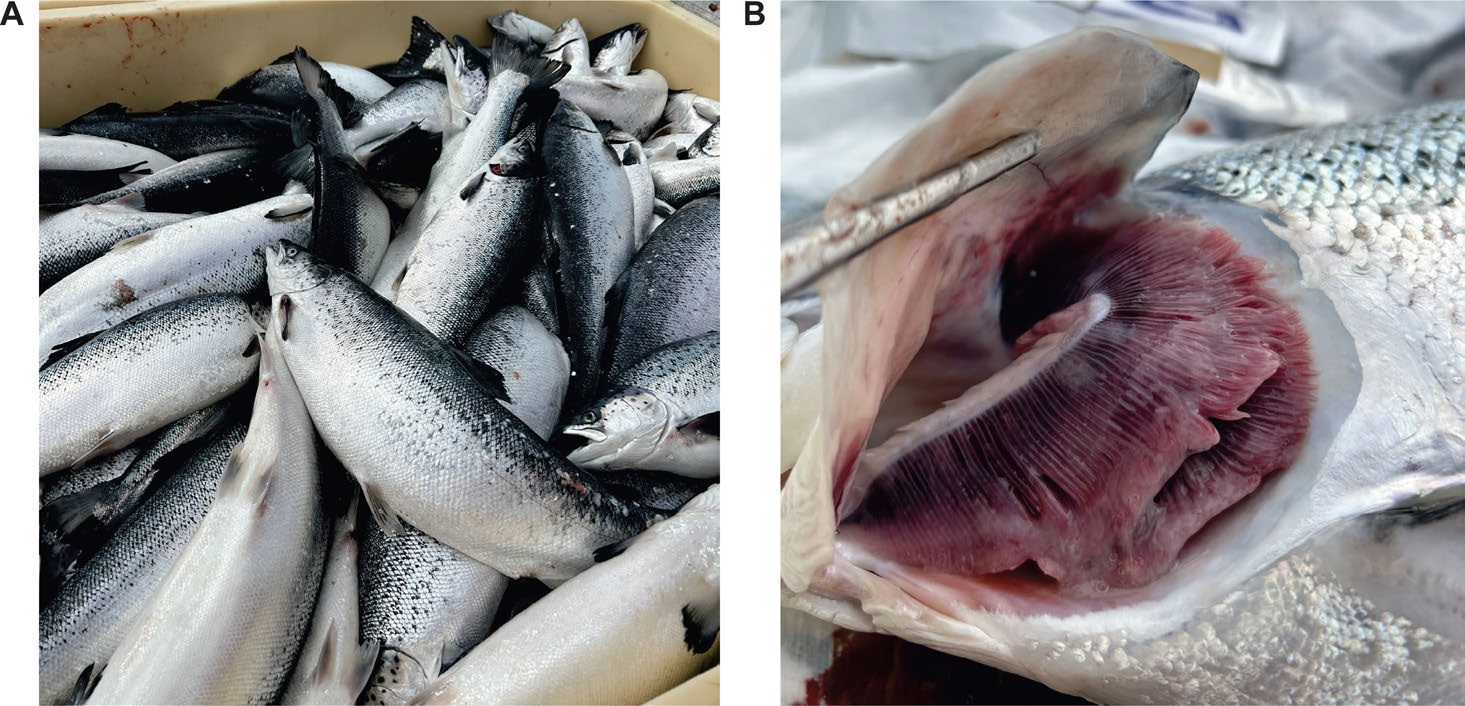
Fish for sampling at Fornes on the 26th of April. (A) Fish with normal external appearance. (B) Gills with excessive amount of mucus. *Photo*: Nora Brække, Nordlaks.

Necropsy was carried out by fish health personnel on the 26th of April on 20 representative moribund fish clearly affected by the algae. The fish were euthanized with an overdose of benzocaine (200 mg/mL, Benzoak vet., STIM AS, Leknes, Norway), 150–200 mL/L in a tank with sea water. The fish were generally observed with a normal external appearance, except for the gills (Figure 8A). Acute pathological gill lesions were observed in seven individuals with excessive mucus on the gills and multifocal petechial haemorrhages (Figure 8B). In three of these fish, chronic gill changes were also evident, presenting as multifocal lamellar thickening appearing pale. Internal examination revealed pathological alterations in five fish, including mottled liver and the presence of blood‐tinted serous fluids in the pericardial‐ and abdominal cavities.

### 2.9 Histological Analysis

Organs for histological analysis were collected on the day abnormal behavior and increased mortality was observed, the 26th of April. 10 of the 20 moribund fish collected for necropsy (see section 2.8 Necropsy of the Fish) were sampled for histological analysis; organs included gills, pseudobranch, heart, liver, pyloric caeca, pancreas, spleen, and muscle. The tissues were immediately fixed in 4% neutral buffered formaldehyde (1:10 ratio) and sent to the Norwegian Veterinary Institute in Harstad for analysis (see Supporting Information for detailed description of the histological method).

**FIGURE 9:**
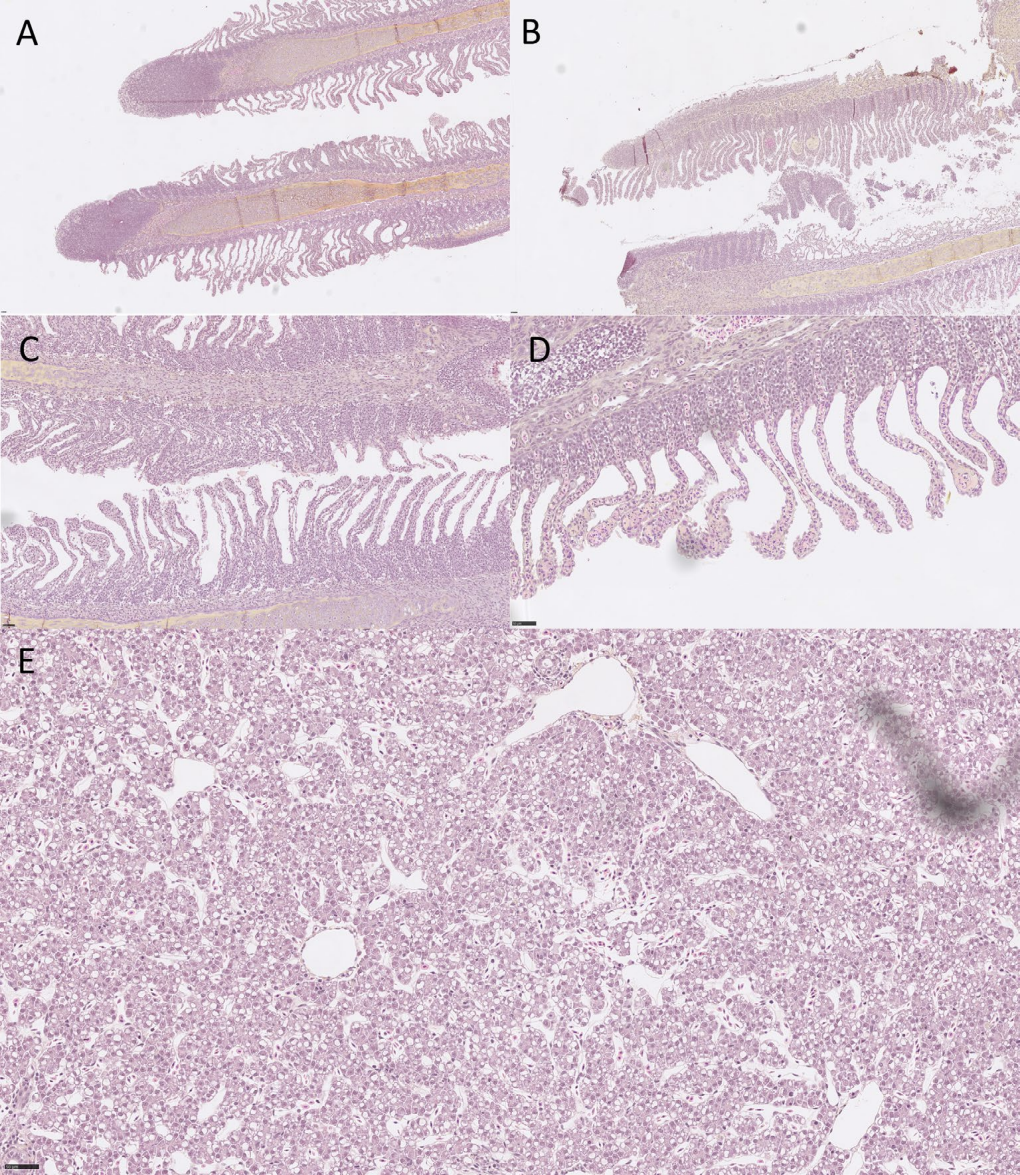
Gill samples from four separate individuals (A–D) and a liver sample (E) collected at Fornes on the 26th of April, Haematoxylin–Eosin‐Saffron (HES) stain, scale 50 μm. The observed tissue changes encompassed: (A) Lamellar fusions, multifocal lamellar haemorrhages and a moderate degree of mucous cell hyperplasia. (B) Moderate to pronounced proliferation of mucous cells and multiple lamellar haemorrhages. (C) Moderate to pronounced proliferation of mucous cells, as well as lamellar fusions, some epithelial proliferation, infiltration of inflammatory cells and necrosis of scattered, single cells in the multilayered epithelium at the basis of the lamellae. (D) Multifocal lamellar haemorrhages, and necrosis of scattered, single cells in the multilayered epithelium at the basis of the lamellae. (E) Diffuse, increased vacuolization of the hepatocytes. Clear, spherical vacuoles, displacing the nuclei, indicating lipid‐type vacuolization. Toxic exposure is one possible causal factor, although not excluding other aetiologies.

Histological examination was done on all sampled organs from the 10 necropsied fish. There were moderate to pronounced tissue changes in the gills (Figure 9A–D). The main findings included necrosis, moderate to pronounced mucus cell proliferation, inflammation, epithelial proliferation with partly fused lamellae, as well as circulatory disturbances with multifocal lamellar haemorrhages. Some of the observed changes indicated algal influence. However, the sum of the observed alterations pointed towards a combination of acute to subacute, as well as more chronic changes. This, seen in combination with the disease history on the site, indicated a complex aetiology. Observations of inflammatory infiltrations in the heart as well as in the red skeletal muscle of some individuals led to a suspicion of heart‐ and skeletal muscle inflammation (HSMI). There were several fish with possible degenerative changes in the liver, such as hepatocyte vacuolization (Figure 9E). Vasculitis/perivascular infiltration of leukocytes was observed in one of these, as well as two additional fish. In the spleen, a modest to moderate degree of circulatory disturbance was observed in several fish. The kidney was affected by nephrocalcinosis in two fish, with other samples displaying signs of circulatory disturbance and/or inflammation. Other organs (pseudobranch, pyloric caeca, and pancreas) had little or no notable pathological changes.

## 3 Discussion

The harmful algal bloom (HAB) at Fornes can be argued to have been predictable, but was experienced as a black swan event; unexpected with significant consequences. In hindsight it can be questioned why the HAB was so unexpected as similar documented major blooms involving related species occurred in the region during the same season in 2019 and 1991, with comparable environmental conditions leading up to the events. On the other hand, during the spring of 2025, it was 34 years since the bloom in 1991 and only 6 years since the bloom in 2019. There may in other words have been an expectation that the next HAB would not reoccur for many years yet. That it was unexpected and unforeseen is illustrated by the fact that, despite the feeding operators already on the 24th of April observed a noticeable decline in fish appetite and that the water had a milky appearance, they did not immediately realise that it was a HAB event. Part of the explanation for this is that such observations are not solely caused by HABs; rather, the contrary. Shorter periods with appetite drops are not uncommon during a production cycle and can have several natural explanations such as changes in the weather, reduced water quality, or other stressful factors such as a whale passing by the farm (Nilsson et al. 2025). Turbid water is also not unusual during the spring, which is the season for the natural spring plankton blooms, which in most cases do not cause any significant harm (Degerlund 2011; Seuthe 2011). After the HAB in 2019, the fish farm company implemented routines and risk management strategies for handling future HABs and, as part of this, performed a risk assessment for a HAB in the area shortly before the incidence at Fornes. Based on the surveillance samples, environmental conditions, fish health and welfare, the risk was considered to be low in the nearest future. This illustrates how unpredictable HABs can be.

Comparing the HABs in 1991, 2019 and 2025 in the region, the environmental conditions prior to the bloom were typically marked by a mild winter, freshwater runoff, sunny weather and little wind (Rey and Aure 1991; Karlsen et al. 2019; Karlson et al. 2021; Fon 2025). Higher concentrations of *C. leadbeateri* were associated with lower salinity, both in the HABs in 1991 and 2019 (Rey and Aure 1991; Karlson et al. 2021; John et al. 2022; Fon 2025). The salinity was not measured at Fornes in 2025, but based on the season, with freshwater runoff, similar findings can be assumed. During the week when Fornes was affected by the HAB, fishermen in the area reported a high number of trees floating in Øksfjorden, possibly originating from a landslide approximately 900 m away from the salmon farm. Hypotheses among the workers at Fornes were that the landslide might have contributed to an increased amount of nutrients in the fjord favouring growth of harmful algae. However, given the limited size of the landslide and the strong water currents in the fjord, it is the authors' judgement that it is rather unlikely that this event had a significant influence on the nutrient conditions in the fjord.

In the Risk Report Norwegian Fish Farming 2026 (Grefsrud et al. 2026), the emission of dissolved nutrients (nitrogen and phosphorus) from open‐mesh aquaculture pens is assessed as a potential driver of eutrophication favouring phytoplankton growth in coastal waters. However, the report finds that at a national level, nutrient emissions from fish farms are currently assessed as low risk for causing widespread eutrophication effects, because modelled increases in phytoplankton production remain well below internationally recognised reference thresholds and are not consistently supported by field measurements. On the other hand, it is also noted that in some defined areas of high production and limited water exchange there may be times of elevated probability for increased production, indicating a need for continued monitoring. For the geographical area where the salmon farm Fornes is located, the report concludes that nutrient emission from salmon farming is low, with model estimates showing only a modest (9.1%) increase in phytoplankton production which is far below the reference level (50%) linked to eutrophication concerns (Grefsrud et al. 2026). To support this, Fornes is also located in a fjord with high currents and at a distance to other salmon farms (Synvis 2025; Norwegian Directorate of Fisheries n.d.‐b).

The HAB at Fornes has many similarities to previous HAB events. These include varying degrees of increased mortality in farmed salmon, lethargic fish swimming in the upper layers of the water column, visually increased respiratory rate, and reduced appetite (Rey and Aure 1991; Treasurer et al. 2003; Mitchell and Rodger 2007; Rodger et al. 2011; Karlsen et al. 2019; Karlson et al. 2021; Esenkulova et al. 2022; John et al. 2022; Fon 2025). Another clear observation at Fornes was seemingly healthy fish, swimming deep in the cage and displaying behaviors indicative of stress. Similar patterns have also been reported at other salmon farms during previous algal blooms (Dale et al. 2023). What puts the HAB at Fornes apart from previous HABs and also the farms later affected in the 2025 HAB is that the initial water samples showed high densities of both *P. pouchetii* and *C. leadbeateri*, where *P. pouchetii* was dominating by order of magnitude and therefore most likely the primary cause of the mortalities the first days. Colonies of *Phaeocystis* spp. form a gel‐like mass in the water columns, acting both as a mechanical barrier and an irritant to the gills, further disrupting oxygen uptake over the gill tissue (Schoemann et al. 2005). *C. leadbeateri*, on the other hand, although not yet fully understood, is believed to produce a neurotoxin and has also demonstrated hemolytic activity (Wang et al. 2024; Fon 2025). Increasing amounts of irradiation have been described to increase the toxicity of *C. leadbeateri* (Fon 2025). This was probably also a factor in why the HAB at Fornes became so deadly.

It is documented that both *Phaeocystis* spp. and *C. leabeateri* can affect the gills in such a way that it has severe consequences for the fish (Schoemann et al. 2005; Wang et al. 2024; Fon et al. 2025). High amounts of mucus production on the gills and multifocal petechial bleedings and/or pale appearing focal thickened spots on the lamella correspond well with what has been found in previous HABs (Mitchell and Rodger 2007; Rodger et al. 2011; Karlson et al. 2021). Histological analysis of the gills at Fornes are also consistent with previous cases, involving necrosis, mucus cell proliferation, inflammation and lamellar haemorrhages of acute‐subacute nature, that have all been described in the literature in relation to HABs (Rodger et al. 2011; Østevik 2022). Findings of chronic nature were also present in several fish. Histopathological analysis revealed hepatocyte vacuolization, where a possible cause for the tissue alteration can be algal toxins. Similar findings in liver tissue have been described in the literature (Esenkulova et al. 2022). However, blood tinted free fluid in the heart cavity has, as far as we are aware, not been previously reported as a pathological finding associated with any HAB, but this was a recurrent observation at several salmon farms affected by the HAB in 2025 according to fish health personnel in the area (personal comment, 2025). On the other hand, at Fornes, circulatory disturbances caused by *Piscine orthoreovirus* (PRV) cannot be ruled out as the cause for this specific pathological observation (Finstad et al. 2014). This also applies to the mottled appearance of the liver (Dalum et al. 2025). During the bloom in 2025, fish without previous gill diseases were also experiencing high mortality at other affected farms (Dalum et al. 2025). Due to the acute nature of certain HAB events it is worth noting that fish can die from HABs without having any observed pathological lesions (Black et al. 1991; Rodger et al. 2011; Østevik 2022). All in all; low mortality in the months leading up to the HAB, with only minor chronic pathological findings present prior to the incidence. In combination with the sudden drop in appetite, changes in behavioural patterns, the presence of specific algal species in water samples and consistent pathological findings, makes it unlikely that any other major factor played a significant role in the rapidly declining fish health and high mortality at Fornes than the HAB.

The HAB in 2025 did not end with the event at Fornes; it was only the beginning. At least 18 salmon farms in the region were affected and resulted in losses of more than 1.9 million salmon (around 5000 t). A few days after the first strike at Fornes, other farms in the same and neighbouring fjords were affected (see Figure A2 for output from particle dispersion modelling between 29th of April to 4th of May to estimate potential spreading of the 2025 algal bloom conducted by IMR). In all the salmon farms experiencing abnormal behaviour and mortality, *C. leadbeateri* was detected in the water and identified as the main causative species for mortality (Naustvoll 2025a; Norwegian Directorate of Fisheries 2025; Mørk 2025). This was in contrast to the events at Fornes, where both *C. leadbeateri* and *P. pouchetii* were present in high numbers.

The HAB at Fornes during the spring of 2025 is now history. Instead, the next question arises: how should we prepare for future blooms? It is no longer a question whether this will occur again, but rather when and where it will occur. Monitoring and early warning systems are of high importance to detect possible harmful algal blooms. There are several methods on the market today such as water samples, satellite observations, numerical modelling, machine learning, and ocean sensors (Zahir et al. 2024). These methods can gather important information; however, each of these methods alone has its own limiting factors. Water samples only measure at a defined point in volume and time and are time‐consuming to analyse; satellite observations are dependent on clear skies; numerical modelling and machine learning are dependent on great amounts of data, and it becomes very expensive to invest in and maintain a sufficient number of devices (Zahir et al. 2024). Zahir et al. (2024) highlight the importance of integrating several methods to optimise forecasting and early warning of HABs. In this particular case, the HAB at Fornes and the HABs occurring after Fornes were most likely a series of local blooms, contrary to the 2019 HAB in the region, which was a larger and continuous bloom spreading with the water masses affecting several fjords and farms (Hoddevik 2025). This also underscores the difficulties in predicting a potential HAB since they seem to vary from time to time even when it is the same algal species. The aquaculture industry is dependent on further research in this field in order to have monitoring systems that are applicable for field conditions, where the results are received in time for the farmer to implement necessary measures.

As has been highlighted several times in this article, adverse consequences of a HAB can manifest rapidly following the initial detection of early indicators, if any warning signs at all. Therefore, measures that can be implemented quickly after identifying the occurrence of a HAB are crucial to minimise the negative impacts for the fish. The most common non‐specific measures are feed withdrawal, minimising handling and stress, early harvest and relocating the fish to another site. However, the latter two require some degree of planning ahead and the optimal decision is also depending on various factors such as the size and health of the fish (Dale et al. 2022). There is shortage of evidence‐based specific measures to implement when a HAB occurs. Instead, some semi‐specific measures are repeatedly mentioned in reports and articles, such as the use of different kinds of shielding skirts, upwelling and bubble curtains (Gallardo‐Rodríguez et al. 2019; Dale et al. 2022; Jónsdóttir et al. 2023; Oh et al. 2023). Various cage technologies are also mentioned as a protective option for the fish against environmental incidents (Dale et al. 2022; Hylin 2025; Madaro et al. 2025). One report noted that perimeter skirts extending 12–15 m may be insufficient, depending on the depth at which the algae occur (Dale et al. 2022). Instead, there are semi‐closed technologies extending deeper, which have shown to be protective for the fish against hydrozoan invasion (Madaro et al. 2025). This technology may function as a physical barrier by keeping fish away from the algae, which has shown to be most concentrated in the upper water levels (Oh et al. 2023). Another alternative may be to place salmon farms at exposed locations where the current quickly changes the water or far from land, even open ocean, where there is less risk for an harmful algal bloom compared to in smaller fjord systems (Hommedal et al. 2019). Lastly, submerged cages are also mentioned to reduce the risk of HABs by keeping the fish deeper than the habitat of the algae (Hylin 2025).

Current knowledge on available monitor‐ and mitigation strategies does not point to a single definite solution suitable for all salmon farms and the possibilities are limited. The event at Fornes highlights the importance of a carefully considered contingency plan with specific measures targeted for a potential HAB for each individual farm site. In this particular case it seemed reasonable to implement feed withdrawal as a first‐line response to further assess the next crucial step in protecting the fish. Raising awareness of changes in the water, fish behaviour, and welfare in risk periods is of high importance to ensure appropriate response without delay. To conclude, the dramatic incidence at Fornes highlights the urgent need for monitoring and early warning systems for HABs, as well as further development of suitable mitigation strategies and contingency plans to minimise the effect of HABs once they have been forecasted or detected.

## Author Contributions

J.S.: conceptualization, data curation, formal analysis, funding acquisition, investigation, methodology, project administration, resources, validation, visualisation, software, writing – original draft, writing – review and editing. M.N.P.: validation, investigation, writing – original draft, writing – review and editing. T.S.: supervision, validation, funding acquisition, writing – original draft, writing – review and editing. P.F.L.: validation, funding acquisition, writing – original draft, writing – review and editing. J.C.S.: validation, visualisation, software, formal analysis, writing – original draft, writing – review and editing. L.‐J.N.: validation, visualisation, software, writing – original draft, writing – review and editing. L.H.S.: conceptualization, investigation, funding acquisition, methodology, formal analysis, validation, visualisation, software, data curation, writing – original draft, writing – review and editing.

## Funding

This work was supported by UiT The Arctic University of Norway and SpareBank 1 Nord‐Norge through HAVNÆR, 2024/299699.

## Conflicts of Interest

The authors declare no conflicts of interest.

## Supporting information


**Figure A1.** Picture taken from a well boat of two of the other well‐boats collecting fish for emergency harvest and three work boats assisting with the crowding process and collecting dead fish.

**Figure A2.** Output from particle dispersion modelling between 29th of April to 4th of May to estimate potential spreading of the 2025 algal bloom conducted by IMR. They used the Norkyst model systems, with current forecasts issued by the Norwegian Meteorological Institute (MET Norway) as input data. The location of the salmon farm Fornes is marked with a red dot. (A) Initial dispersion of particles at 1 m depth (29th of April). (B) Predicted dispersion after 48 h (1st of May). (C) Predicted dispersion after 96 h (3rd of May). (D) Predicted dispersion after 132 h (5th of May). (E) Predicted dispersion after 156 h (6th of May). (F) Predicted dispersion after 180 h (7th of May).

**Data S1:** jfd70162‐sup‐0003‐DataS1.docx.

## Data Availability

The data that support the findings of this study are available from the corresponding author upon reasonable request.
