# Supplementary figures and images for: First Strike: Description of the Events at the First Salmon Farm Affected by the 2025 Algal Bloom in Northern Norway
Source: J Fish Dis. 2026 Mar 15;49(8):e70162. doi: 10.1111/jfd.70162 (PMC13331527; doi:10.1111/jfd.70162)

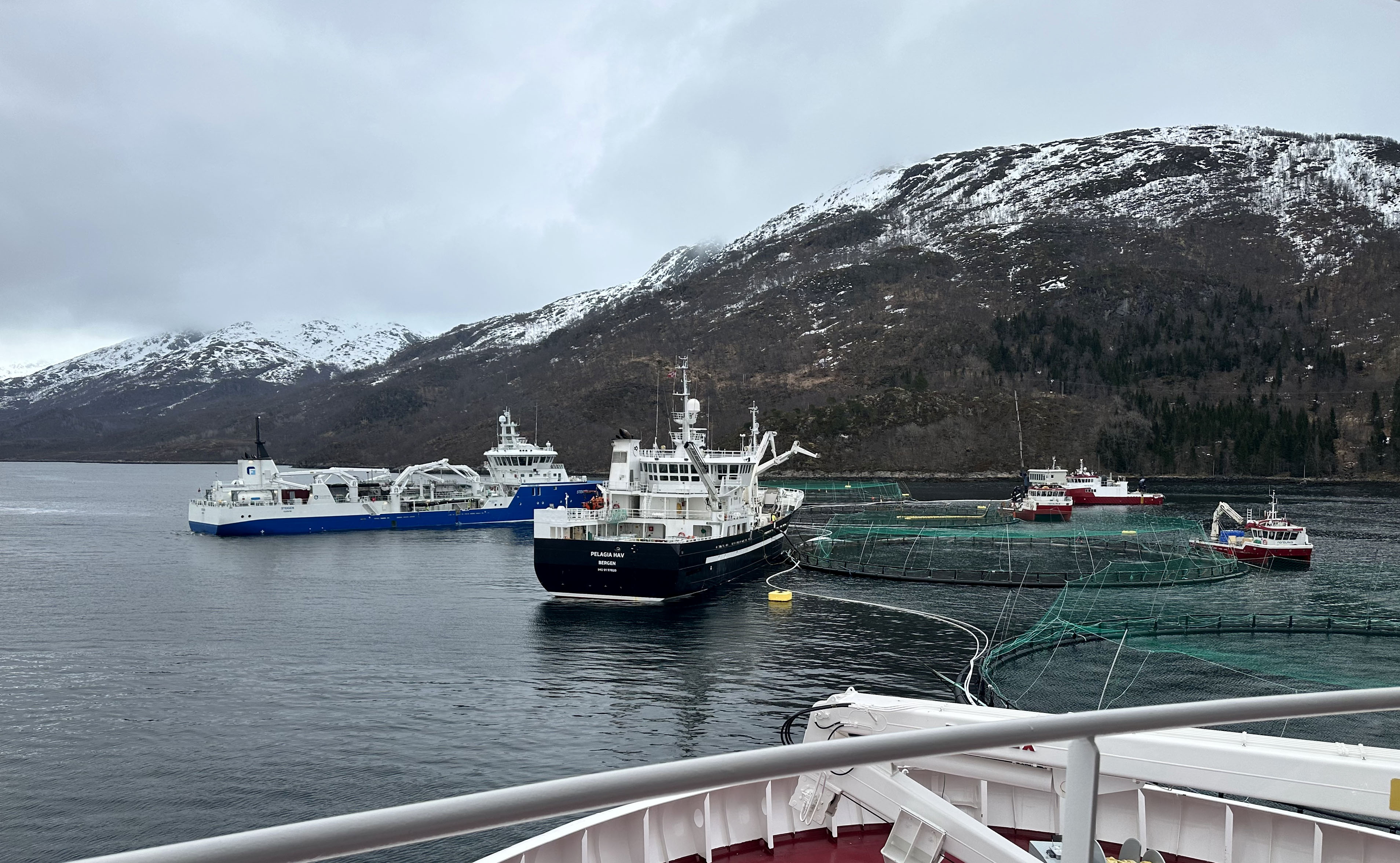

Supplement: Supplementary file 1 — Figure A1. Picture taken from a well boat of two of the other well‐boats collecting fish for emergency harvest and three work boats assisting with the crowding process and collecting dead fish. [file JFD-49-e70162-s002.jpg]

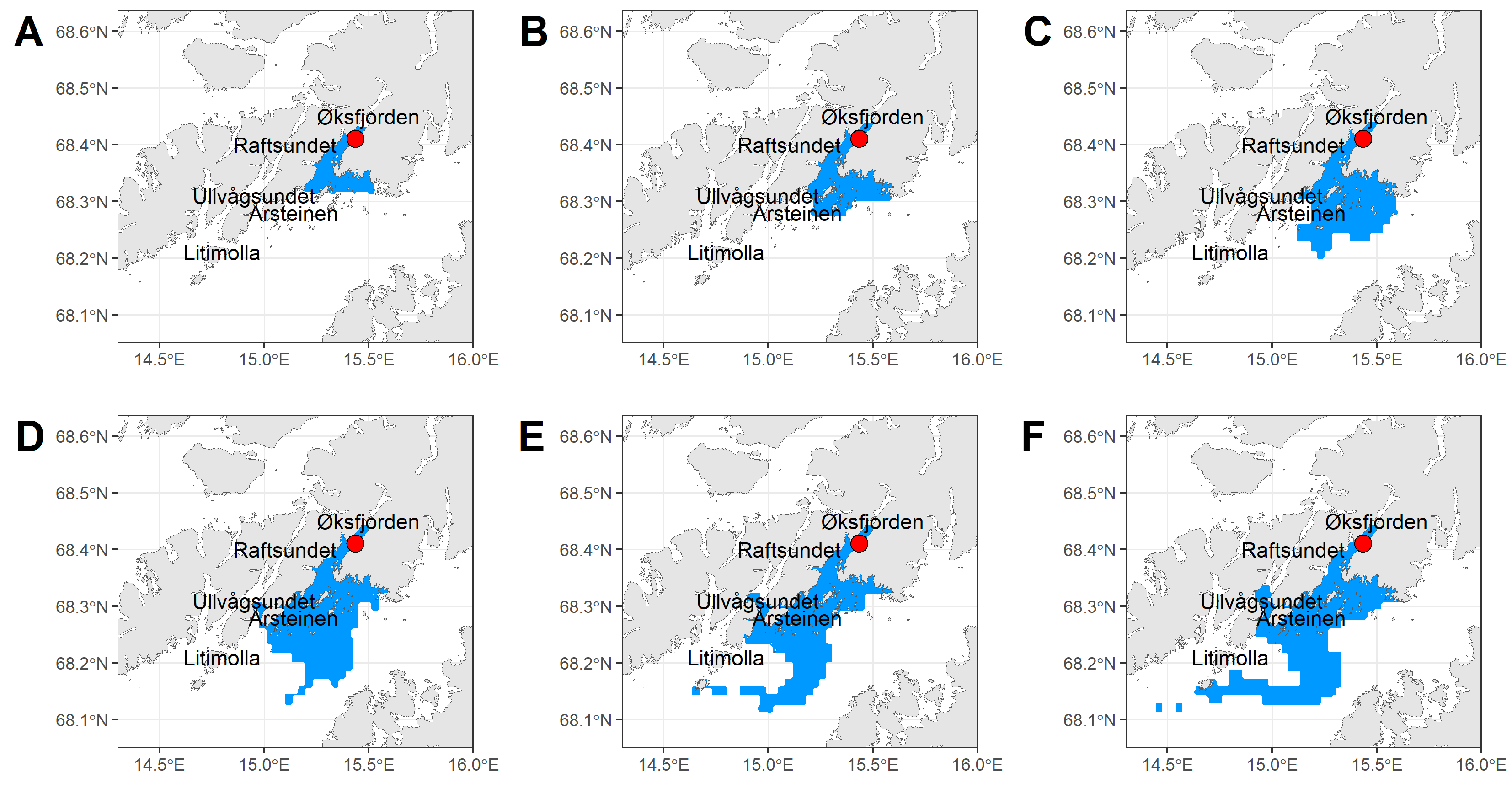

Supplement: Supplementary file 2 — Figure A2. Output from particle dispersion modelling between 29th of April to 4th of May to estimate potential spreading of the 2025 algal bloom conducted by IMR. They used the Norkyst model systems, with current forecasts issued by the Norwegian Meteorological Institute (MET Norway) as input data. The location of the salmon farm Fornes is marked with a red dot. (A) Initial dispersion of particles at 1 m depth (29th of April). (B) Predicted dispersion after 48 h (1st of May). (C) Predicted dispersion after 96 h (3rd of May). (D) Predicted dispersion after 132 h (5th of May). (E) Predicted dispersion after 156 h (6th of May). (F) Predicted dispersion after 180 h (7th of May). [file JFD-49-e70162-s001.tiff]
