# Supplementary material for: First Strike: Description of the Events at the First Salmon Farm Affected by the 2025 Algal Bloom in Northern Norway
Source: J Fish Dis. 2026 Mar 15;49(8):e70162. doi: 10.1111/jfd.70162 (PMC13331527; doi:10.1111/jfd.70162)
Supplement: Supplementary file 3 — Data S1: jfd70162‐sup‐0003‐DataS1.docx. [file JFD-49-e70162-s003.docx]

**Histology method**

Tissue samples were collected from 10 individuals. The samples were taken from the gills, pseudobranch, skin and skeletal muscle, heart, kidney, liver, spleen and pyloric caecae/pancreas.

The tissue samples were immediately fixed in 4% neutral buffered formaldehyde 270 (1:10 ratio) and sent to the Norwegian Veterinary Institute (NVI) in Harstad, Norway for further processing and analysis.

The samples were processed by histotechnologists at NVI according to standard histological procedures, including dissection, fixation, paraffin tissue processing, paraffin embedding, sectioning, and staining.

Processing comprised dehydration, clearing, and paraffin infiltration, and was performed using a Milestone Logos tissue processor. Following processing, the Leica EG 1150 embedding station was used to embed the samples.

Sectioning was carried out through the application of a Leica RM2255 rotary microtome. Final sections were cut at a thickness of 2.5 µm or 3 µm and mounted on glass slides.

The sections were stained using the hematoxylin–eosin–saffron (HES) method with an automated staining system (Sakura Tissue-Tek Prisma Plus). Reagents were prepared in accordance with the laboratory’s standard operating protocols.

Step by step procedure for HES-staining:

1. Deparaffinizing with xylene (0:35 + 4:30 + 5 minutes)

2. Rehydration with absolute alcohol (2:30 + 2:30 minutes)

3. Rehydration with 95% alcohol (45 seconds)

4. Rehydration with 70% alcohol (45 seconds)

5. Rinsing in running tap water (2 minutes)

6. Incubation hematoxylin solution (Gill 2) (3 minutes)

7. Rinsing in running tap water (2 minutes)

8. Blueing of slides by dipping them in ammonia water (1 minute)

9. Rinsing in running tap water (2 minutes)

10. Hydrochloric acid alcohol (5 seconds)

11. Rinsing in running tap water (30 seconds)

12. Incubation in eosin solution (0,5% aq) (45 seconds)

13. Rinsing in running tap water (40 seconds)

14. Quick rinse in 95% alcohol to remove excess eosin (20 seconds)

15. Dehydrate through absolute alcohol (2 minutes)

16. Incubation in saffron solution (Masson, 50:50 with absolute alcohol). (15 seconds)

17. Dehydration with absolute alcohol (15 + 20 seconds)

18. Xylene (30 seconds)

Object glasses were coverslipped in a Sekura Tissue Tek Film™️ coverslipper.

Histological sections were digitized using a Hamamatsu NanoZoomer S360 slide scanner with NanoZoomer NZAcquire software (version 3.2.0). Scanning was performed at 20× or 40× magnification.

Histopathological evaluation was performed using the NDP.view software.
